# SARS-CoV-2 infection is associated with an increase in new diagnoses of schizophrenia spectrum and psychotic disorder: A study using the US national COVID cohort collaborative (N3C)

**DOI:** 10.1371/journal.pone.0295891

**Published:** 2024-05-30

**Authors:** Asif Rahman, Michael Russell, Wanhong Zheng, Daniel Eckrich, Imtiaz Ahmed, On behalf of the N3C Consortium

**Affiliations:** 1 Department of Industrial & Management Systems Engineering, West Virginia University, Morgantown, WV, United States of America; 2 School of Medicine, West Virginia University, Morgantown, WV, United States of America; 3 Nemours Children’s Health, Jacksonville, FL, United States of America; Universitas Pelita Harapan, INDONESIA

## Abstract

Amid the ongoing global repercussions of SARS-CoV-2, it is crucial to comprehend its potential long-term psychiatric effects. Several recent studies have suggested a link between COVID-19 and subsequent mental health disorders. Our investigation joins this exploration, concentrating on Schizophrenia Spectrum and Psychotic Disorders (SSPD). Different from other studies, we took acute respiratory distress syndrome (ARDS) and COVID-19 lab-negative cohorts as control groups to accurately gauge the impact of COVID-19 on SSPD. Data from 19,344,698 patients, sourced from the N3C Data Enclave platform, were methodically filtered to create propensity matched cohorts: ARDS (n = 222,337), COVID-19 positive (n = 219,264), and COVID-19 negative (n = 213,183). We systematically analyzed the hazard rate of new-onset SSPD across three distinct time intervals: 0-21 days, 22-90 days, and beyond 90 days post-infection. COVID-19 positive patients consistently exhibited a heightened hazard ratio (HR) across all intervals [0-21 days (HR: 4.6; CI: 3.7-5.7), 22-90 days (HR: 2.9; CI: 2.3 -3.8), beyond 90 days (HR: 1.7; CI: 1.5-1.)]. These are notably higher than both ARDS and COVID-19 lab-negative patients. Validations using various tests, including the Cochran Mantel Haenszel Test, Wald Test, and Log-rank Test confirmed these associations. Intriguingly, our data indicated that younger individuals face a heightened risk of SSPD after contracting COVID-19, a trend not observed in the ARDS and COVID-19 negative groups. These results, aligned with the known neurotropism of SARS-CoV-2 and earlier studies, accentuate the need for vigilant psychiatric assessment and support in the era of Long-COVID, especially among younger populations.

## Introduction

It has been over three years since the initial identification of SARS-CoV-2 infection (hereafter referred to as COVID-19)in the USA. Despite the development of a vaccine and efforts to combat the pandemic, there are still many unanswered questions. Particularly, the long-term effects of COVID-19 on mental health are yet to be fully unwrapped and associated with the disease. Several preliminary studies [1–3] have suggested an increased risk of mental illness following a COVID-19 diagnosis, including but not limited to anxiety, depression, mood disorder, post-traumatic stress disorder (PTSD), insomnia, dementia, delirium, encephalitis, psychosis, and nerve disorder. It is important to note that viral infections resulting from recent outbreaks of severe acute respiratory syndrome (SARS) in 2002 and Middle East respiratory syndrome (MERS) in 2012, both caused by coronavirus closely related to SARS-CoV-2, were also associated with neurological manifestations in some cases [3].

COVID-19 has a multi-organ pathology that includes the human brain and the central nervous system [4]. It has been detected both in the brain and cerebrospinal fluids of the diagnosed patients. The COVID-19 patients show a greater cognitive decline compared to the non-COVID patients. It has also been associated with brain structural change [5]. Recent studies have suggested that more than one-third of the infected individuals develop neurological symptoms in the acute phase of the disease, and around 34% of them show brain abnormalities [6, 7]. COVID-19 has been linked to excessive and dysregulated immune responses that can lead to systemic inflammation. Patients with severe COVID-19 have been found to have elevated levels of various inflammatory markers in their blood, such as C-reactive protein (CRP), ferritin, interleukin-6 (IL-6), and others. Among them, higher levels of CRP and IL-6 are linked to a higher risk of different neurological conditions such as major depressive disorders.

It has been well established that a bidirectional interaction exists between the central nervous system, specifically the brain, and systemic inflammation [8]. In the brain, microglia are the principal cells involved in modulating the effects of remote inflammatory stressors resulting in neuro-inflammatory manifestations of systemic processes arising from multiple causes (trauma, infection, auto-immune processes, etc.) [9]. Microglial functional and structural alterations have been found in multiple major psychiatric disorders [9–11] although the presence, degree, and configuration of such alterations vary between diagnoses. Therefore, the etiologic and therapeutic significance of these observations remains unclear [12].

A growing body of literature has implicated systemic inflammation associated with critical illness in the development of delirium [13, 14]. In turn, the occurrence of delirium during critical illness is associated with persistent deficits in neurocognitive function following survival [15]. The presence of pre-exposure decline in cognitive function is associated with an increased risk of post-critical illness persistent neurocognitive disability [16, 17]. Differences in epigenetic DNA methylation patterns in critically ill patients with and without delirium have recently been reported [18]. It is now well established that systemic inflammation affects brain function in critical illness and that these effects are persistent beyond the intensive care episode [19, 20]. While the exact mechanisms are still incompletely characterized, epigenetic modification of DNA directed protein transcription may play a potential role.

Major psychiatric illness is known to have a strong genetic component. However, variable penetrance suggests that environmental factors are also important in the development of clinical disease [21]. Emerging data suggests a significant association between neuroinflammatory changes and major psychiatric illnesses. It has long been speculated that viral infectious illness may have a causative relationship to major psychiatric illness [22–24], especially early in development [25, 26]. The strongest associations to date between inflammatory and infectious illness and major psychiatric disease involve the development of schizophrenia, bipolar disorder, and major depression [9, 10, 27]. These associations trace back to the 1918 flu pandemic, which led to the viral hypothesis of schizophrenia. This hypothesis suggests that viral pandemics could result in an increase in SSPD [24].

Given the increasing evidence demonstrating a link between systemic inflammation generally and viral illness specifically, and subsequent neurocognitive function, the role of neuroinflammation in manifesting major psychiatric disorders and the systemic inflammatory effects of COVID-19, the authors sought to establish if COVID-19 could lead to a rise in the onset of significant psychiatric conditions. We decide to concentrate on the schizophrenia spectrum and psychotic disorders (SSPD), as the data linking inflammatory conditions with the emergence or progression of the disease is most compelling in this context.

We are aware that several studies [2, 3, 28] tried to establish an association between COVID-19 and psychiatric manifestations. However, a significant portion of these studies lacked an appropriate comparison group, leading to an incomplete understanding of the incidence and prevalence of neuro-psychiatric disorders in COVID-19 patients. To address this limitation, our study incorporated a control group comprising individuals with Acute Respiratory Distress Syndrome (ARDS) and those who tested negative for COVID-19. This approach ensured a more precise correlation between COVID-19 and SSPD. Furthermore, we leveraged the N3C platform, utilizing its vast, robust, and long-term data set to effectively discern and quantify the impact of COVID-19 on SSPD.

## Materials and methods

This study received written ethical approval from the West Virginia University (WVU) Institutional Review Board (IRB) under protocol number 2201509313. The study qualified under the WVU Flexibility Review Model, as it involves minimal risk and adheres to the Belmont Report’s ethical principles. Approval was granted on April 12, 2022. Our study was a retrospective cohort study. All data was collected from the National COVID-19 Cohort Collaborative (N3C) Data Enclave platform. The dataset was retrieved on May 31, 2023, and we limited our analysis to include only records up to that date. Throughout the process of data collection and subsequent analysis, the authors did not have access to any information that could be used to identify individual participants.

### Study design and data collection

**Fig 1:**
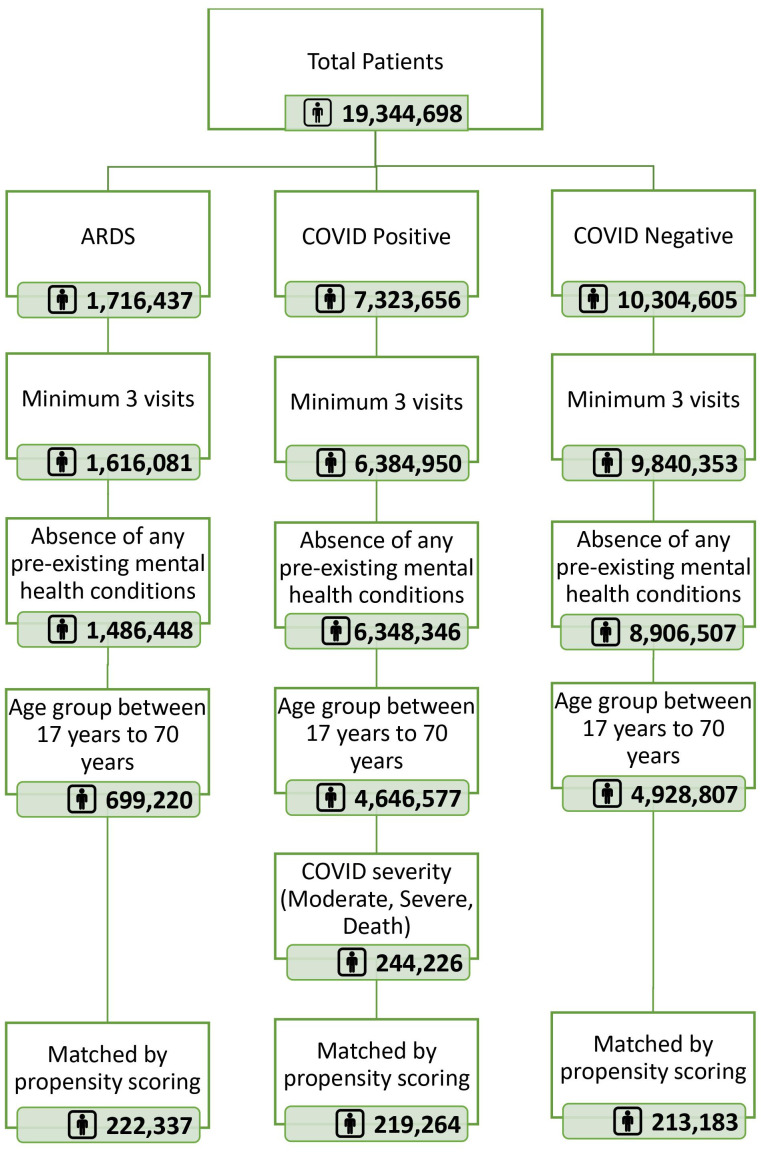
Final cohort selection by applying exclusion criteria.

To achieve our objectives, we initiated a systematic filtering process as depicted in Fig 1. Out of an initial dataset of 19 million patients, we categorized them into three primary groups: ARDS, COVID-19 positive, and COVID-19 negative. We applied specific criteria to refine these cohorts. Firstly, we considered only those with a minimum of three visits. Secondly, we excluded patients with any pre-existing mental health conditions and further narrowed down our scope to individuals aged between 17 and 70 years. Notably, within the COVID-19 positive group, we focused on patients characterized by moderate, severe, or terminal outcomes due to the virus. After implementing these filters, our COVID-19 positive cohort size was finalized at 244,226. To ensure that our data from these groups could be directly compared, we also implemented a propensity score matching technique.

### Cohorts

Three distinct cohorts were constructed for this study: one case and two controls. The case cohort was made up of patients diagnosed with COVID-19, based on the N3C defined computable phenotype version 4.0. [29]. In order to derive meaningful insights, we limited our COVID-19 positive patients to those with moderate to severe manifestations. This categorization was determined by several factors, including the duration of inpatient hospital stays, usage of invasive ventilation, application of extracorporeal membrane oxygenation (ECMO), and even unfortunate fatalities.

For our controls, we selected patients diagnosed with ARDS post-January 1st, 2020 but without any record of a COVID-19 diagnosis during the pandemic. The third group consisted of those who tested negative for COVID-19 and had no prior history of either COVID-19 or ARDS. The starting population for our study needed to have a history of at least three medical visits spanning 365 days or more. To standardize the timeline across cohorts, index dates were determined based on the earliest date of relevant diagnosis or lab test results.

We excluded individuals below 17 years or over 70 years at the time of the index date. Furthermore, patients with the following mental health disorders prior to the index date were also removed from consideration:

- Schizophrenia Spectrum Disorders
- Bipolar Disorders
- Major Depression
- Personality Disorders
- Trauma

For homogeneity, all cohorts underwent 1:1 nearest neighbor (NN) matching on propensity scores using the R “MatchIt” package. This matching was based on the following attributes:

- Gender
- Race
- Ethnicity
- Age groups
- Prior psychiatric drug prescription or administration
- Prior Hypothyroidism diagnosis
- Prior Anxiety diagnosis
- Prior Substance Abuse diagnosis
- Prior Insomnia diagnosis

Please refer to the S1 Appendix for specific Observational Medical Outcomes Partnership (OMOP) codesets [30] used for identifying these attributes. Post-matching, the cohort sizes were 219,264 for COVID-19 positive patients, 213,183 for the lab negatives, and 222,337 for ARDS patients.

### Outcome

Once the cohorts were built, we looked at the first incident post-index date of SSPD. The code sets were developed using the OMOP concept and concept_ancestor tables [30] and reviewed by subject matter experts. While psychiatry continued to debate over the relationship between psychotic symptoms and mood symptoms [31, 32], in this study, to focus on the hallmark thought symptoms characteristic of schizophrenia (such as delusions, hallucinations, or disorganized speech), we separated SSPD from bipolar and depressive disorders. Therefore, while acute psychotic disorder, schizophreniform, schizophrenia, schizoaffective disorders, and related ICD-10 diagnoses were included in the SSPD category, mood disorders with psychotic features were counted in the latter two categories and thus excluded from the analysis.

The data was subsequently structured to include three pivotal columns: a boolean flag denoting whether a patient was diagnosed with SSPD after the index date, the exact date of the patient’s initial SSPD diagnosis post the index date, and the duration in days between the index date and this diagnosis. This data arrangement culminated in a matrix with a single record for each patient, encompassing cohort identifiers, demographic details, other relevant covariates, and the critical outcome variable.

### Statistical analysis

To examine the association between COVID-19 and SSPD, we performed a comparative analysis among the matched cohorts using the N3C platform in R (version 4.0). Our primary predictor variable is the disease type, categorized into three groups: COVID-19 positive, COVID-19 negative, and ARDS. The outcome variable is binary, indicating either SSPD or non-SSPD.

We utilized the Cox Proportional Hazard Model [33] to derive the hazard ratios (HR) of COVID-19 positive patients relative to the COVID-19 negative and ARDS patients. The time-to-event for patients diagnosed with SSPD was measured from their SSPD diagnosis date up to the COVID-19 and ARDS reference dates, which include the dates of their positive COVID test, negative COVID lab test, and ARDS diagnosis. For patients without an SSPD diagnosis, this duration was taken from their most recent recorded visit to the reference date of either their COVID-19 or ARDS diagnosis.

Before deploying the Cox model, it was imperative to test the proportional hazard assumption. In doing so, the Schoenfeld residual analysis, a conventional diagnostic tool for this purpose [34], yielded a significant p-value. This necessitated rejecting the null hypothesis of a uniform proportional hazard over the comprehensive time frame of 180 days. Consequently, we segmented the cohort into three distinct time intervals: 0–21 days, 22–90 days, and beyond 90 days. These intervals were subsequently validated for the proportional hazard assumptions, and the Cox model was then applied to each to ascertain the hazard ratio (HR).

In tandem with the Cox model, we also conducted the Cochran Mantel Haenszel Test [35], the Likelihood Ratio Test [36], the Wald Test [35], and the Log-rank Test [37] across the three-time intervals. A p-value threshold of 0.05 served as the determinant for statistical significance in all these tests.

## Results

The results shed light on the potential long-term psychiatric implications of SARS-CoV-2 infection. The study found robust evidence linking SARS-CoV-2 infection to an augmented risk of Schizophrenia Spectrum and Psychotic Disorders (SSPD). Specifically, COVID-19 positive patients displayed almost double the incident rate (0.56%) compared to COVID-19 negative (0.33%) and ARDS (0.29%) patients, as showcased in Table 1.

**Table 1:** Characteristics of cohorts.

Characteristics	ARDS		COVID-19 Positive		COVID-19 Negative	
	N = 222337		N = 219264		N = 213183	
	Count	Percentage	Count	Percentage	Count	Percentage
**Gender**:						
Female	105175	47.30%	103528	47.20%	105959	49.70%
Male	117162	52.70%	115736	52.80%	107196	50.30%
Other or Unknown	0	0.00%	0	0.00%	28	0.01%
**Age Groups**:						
21 and Younger	7896	3.55%	7449	3.40%	7840	3.68%
22 to 29	19885	8.94%	19160	8.74%	19644	9.21%
30 to 39	31070	14.00%	30298	13.80%	29400	13.80%
40 to 49	35871	16.10%	34407	15.70%	31594	14.80%
50 to 59	52529	23.60%	53059	24.20%	51115	24.00%
60 and Older	75086	33.80%	74891	34.20%	73590	34.50%
**Race**:						
Asian	5519	2.48%	5580	2.54%	5568	2.61%
Black/African American	48251	21.70%	47047	21.50%	45269	21.20%
White/Caucasian	132914	59.80%	126061	57.50%	127741	59.90%
Unknown	29357	13.20%	31810	14.50%	27014	12.70%
Nothing mentioned	1746	0.79%	2701	1.23%	2758	1.29%
Other	4550	2.05%	6065	2.77%	4833	2.27%
**Ethnicity**:						
Hispanic or Latino	34320	15.40%	38196	17.40%	31002	14.50%
Not Hispanic or Latino	171609	77.20%	165071	75.30%	166149	77.90%
Missing/Unknown	16408	7.38%	15997	7.30%	16032	7.52%
**Any Psychiatric Disease (before index date)**:						
No	145612	65.50%	138029	63.00%	137490	64.50%
Yes	76725	34.50%	81235	37.00%	75693	35.50%
**Anxiety Disorder**:						
No	198285	89.20%	201933	92.10%	192958	90.50%
Yes	24052	10.80%	17331	7.90%	20225	9.49%
**Insomnia Disorder**:						
No	211921	95.30%	212449	96.90%	204869	96.10%
Yes	10416	4.68%	6815	3.11%	8314	3.90%
**Hypothyroidism Disorder**:						
No	206762	93.00%	204850	93.40%	198391	93.10%
Yes	15575	7.01%	14414	6.57%	14792	6.94%
**Hypothyroidism Disorder (Lab Test)**:						
No	212801	95.70%	208228	95.00%	201818	94.70%
Yes	9536	4.29%	11036	5.03%	11365	5.33%
**Drug Abuse / Addiction Disorder**:						
No	189594	85.30%	190323	86.80%	188360	88.40%
Yes	32743	14.70%	28941	13.20%	24823	11.60%
**Drug Abuse / Addiction Disorder (Lab Test)**:						
No	218686	98.40%	211424	96.40%	209335	98.20%
Yes	3651	1.64%	7840	3.58%	3848	1.81%
**All Mental Health**:						
No	207448	93.30%	203123	92.60%	197204	92.50%
Yes	14889	6.70%	16141	7.36%	15979	7.50%
**SSPD**:						
No	221700	99.70%	218035	99.40%	212476	99.70%
Yes	637	0.29%	1229	0.56%	707	0.33%
**Bipolar Disorder**:						
No	214506	96.50%	210281	95.90%	205014	96.20%
Yes	7831	3.52%	8983	4.10%	8169	3.83%
**Personality Disorder**:						
No	222034	99.90%	218951	99.90%	212889	99.90%
Yes	303	0.14%	313	0.14%	294	0.14%
**Depression**:						
No	210088	94.50%	205253	93.60%	199865	93.80%
Yes	12249	5.51%	14011	6.39%	13318	6.25%
**Trauma**:						
No	219432	98.70%	217107	99.00%	210308	98.70%
Yes	2905	1.31%	2157	0.98%	2875	1.35%

Using the Cox model, a marked difference in the hazard ratio of new-onset psychiatric outcomes became evident between COVID-19 positive patients and the cohorts of ARDS and COVID-19 lab negative patients (Please refer to Table 2). For all time intervals considered, COVID-19 negative patients were the benchmark for hazard ratio computations. In the immediate 21 days following exposure, the hazard ratio for COVID-19 positive patients was notably high (HR: 4.6; 95% CI: 3.7 to 5.7) when contrasted with ARDS patients (HR: 0.73 CI: 0.53 to 0.99). This suggests that, during the acute phase, individuals positive for COVID-19 were significantly more likely to be diagnosed with SSPD than both their COVID-19 negative and ARDS counterparts.

**Table 2:** Hazard ratios with 95% Confidence Interval (CI) for SSPD in different time frames.

Time Frame (Days)	COVID-19 Negative				COVID-19 Positive				ARDS		
	Hazard Ratio	95% CI			Hazard Ratio	95% CI			Hazard Ratio	95% CI	
		Lower	Upper			Lower	Upper			Lower	Upper
**0–21**	*	*	*		4.6	3.7	5.7		0.73	0.53	0.99
**22–90**	*	*	*		2.9	2.3	3.8		1.1	0.79	1.43
**Beyond 90**	*	*	*		1.7	1.5	1.9		0.97	0.86	1.47

*COVID-19 Negative patient group is considered as references.

**Fig 2:**
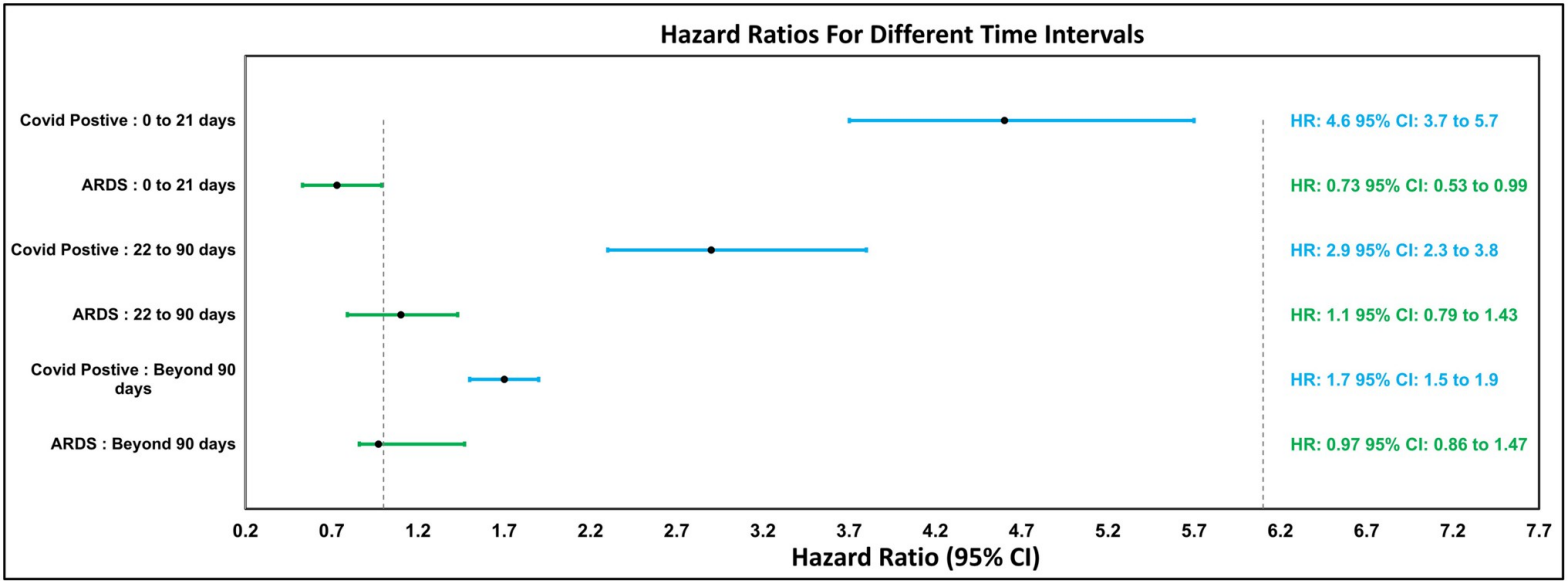
SSPD hazard ratio comparisons.

In the subsequent interval of 22–90 days, the hazard ratio for COVID-19 positive patients remained elevated (HR: 2.9; 95% CI: 2.3 to 3.8), and interestingly, ARDS patients exhibited their peak hazard ratio of the study (HR:1.1, CI: 0.79 to 1.43). Beyond 90 days, the hazard ratio for the COVID-19 positive group experienced a reduction (HR: 1.7; 95% CI: 1.5 to 1.9) yet was consistently higher than that of the ARDS patients (HR:0.97, CI: 0.86 to 1.47). Despite the reduction in hazard ratios as time progressed, it’s salient to note that COVID-19 survivors remain at a heightened risk for SSPD well beyond the immediate aftermath of their infection. The hazard ratios for different time intervals are visually summarized in Fig 2.

Following the detailed hazard ratio analysis, several statistical tests (Please refer to Table 3) were conducted to further validate the findings. The Schoenfeld residuals rest returned a p-value exceeding 0.05, suggesting the retention of the null hypothesis that the Hazard Ratio is consistent over time. Additionally, tests such as the Cochran Mantel Haenszel Test, Likelihood Ratio Test, Wald Test, and Log-rank Test consistently showed p-values less than 0.05, reinforcing a significant association between being COVID-19 positive and receiving an SSPD diagnosis.

**Table 3:** P value for different tests in different time frames.

P Value	Time Frame (In Days)		
	0–21	22–90	Beyond 90
Schoenfeld Residuals Test	0.24	0.75	0.15
Cochran Mantel Haenszel Test	< 0.05	< 0.05	< 0.05
Likelihood Ratio Test	< 0.05	< 0.05	< 0.05
Wald Test	< 0.05	< 0.05	< 0.05
Logrank Test	< 0.05	< 0.05	< 0.05

In our efforts to determine potential demographic factors influencing SSPD occurrence among COVID-19 positive patients, several key demographics emerged as more susceptible to SSPD following a COVID-19 diagnosis. Specifically, males, individuals aged 21 or younger, those of African American descent, and non-Hispanic or Latino individuals showcased a heightened vulnerability (see Tables 4 and 5 in the S1 Appendix for details). Intriguingly, these same demographic trends, with the exception of the age factor, were mirrored in the SSPD occurrence among both the COVID-19 negative and ARDS groups (refer to Tables 6 and 7 in the S1 Appendix for further insights). While these findings undoubtedly warrant a deeper exploration, we strongly advocate for future research endeavors to prioritize this critical observation regarding the younger people being more susceptible to post-COVID SSPD.

## Discussion

Our study indicates that the likelihood of developing SSPD after a COVID-19 infection is higher than in ARDS and COVID-19-negative patients. The significance of various demographic factors has also emerged from our results. These insights underscore the vital importance of keeping a close watch on the mental well-being of those recovering from COVID-19. Their persistent increased risk points to a wider societal concern, especially regarding severe psychiatric conditions like SSPD.

Extensive literature has accumulated since 2000 that indicates an association between various inflammatory markers, changes in the structure and function of a variety of cellular components of the brain, and the development of major psychiatric illnesses. No longer thought to be “immunologically privileged” by virtue of the blood-brain permeability barrier, it is now well established that the brain is extensively influenced by systemic inflammation and, in turn, can modulate systemic inflammation through descending [38] and biochemical [39] pathways. Inflammatory influences in the brain are structural [40] and functional [8]. Effects of maternal development on in-utero brain development and subsequent offspring behavior have been demonstrated in animal models [41] although the relevance to mental illness in humans remains to be determined. Inflammatory influences on the developed brain have also been demonstrated and tied to clinically relevant behavior [42–44]. However, studies evaluating the relationship between various inflammatory markers and clinically relevant behavior have yielded inconsistent results [13, 14, 45–47]. At the moment, these inflammatory changes cannot be causally linked directly to specific conditions, but they do provide potential mechanistic insights and may become plausible therapeutic targets in conditions where the response to currently available medications varies widely [14, 40, 43].

With that background, our group sought to establish whether any association existed between SARS-CoV-2 infection and the extensive accompanying inflammatory response and new-onset psychiatric illness. Our results are consistent with the hypothesis that COVID-19 infection and the accompanying inflammatory state (“cytokine storm”) is positively associated with the new onset of SSPD. Further, these results strongly suggest a direct relationship between the development of SSPD and the severity of the disease state and presumably the intensity of the attendant inflammatory response. We acknowledge the viewpoint [48] that the increase in mental health problems during the COVID-19 pandemic may not stem directly from the virus itself but from indirect consequences of the pandemic, such as isolation, unemployment, and other stress-related factors. It is plausible that receiving a COVID-19 diagnosis, particularly during the pandemic’s early days, could result in considerable psycho-social stress, known to be a risk factor for SSPD. However, in our analysis (see Table 8 in the S1 Appendix) we have found that the elevated risk of developing SSPD remained consistent over different time periods, from the pandemic’s onset in 2020 through to 2022, a period marked by the availability of vaccines and a gradual return to normalcy. This consistency suggests there might be an underlying link beyond mere psycho-social stress. Our findings also align with previous research that found a higher occurrence of retroviral infection markers in the cerebral spinal fluid and brain tissue of schizophrenia patients compared to non-schizophrenic controls with and without the noninflammatory neurologic disease [49]. With the demonstrated neurotropism of the SARS-CoV-2 virus [50, 51], our results strongly support a potentially causal relationship between COVID-19 infection and new onset SSPD. Long-COVID syndrome, known for causing mild cognitive issues, has been explored for its psychiatric effects. However, smaller studies have yet to establish a clear connection between long-COVID and the onset of major psychiatric conditions [52]. It’s important to note that our study did not aim to investigate this specific relationship.

The strengths of our study are underscored by the utilization of an expansive and meticulously curated national dataset, enabling an in-depth analysis of a vast number of records. Furthermore, our rigorous approach to defining exclusionary criteria and cohort matching bolster the robustness of our findings. However, the study does possess notable limitations. Foremost among these is, same as any other retrospective studies, the dependence on documented diagnoses (ICD-10 coding) to pinpoint new instances of SSPD. It is conceivable—perhaps even probable—that certain patients might have been inaccurately diagnosed, thereby skewing their categorization as determined by our data extraction methodology. The markedly elevated hazard ratio observed during the acute (0–21 day) phase, for instance, is challenging to rationalize, given that definitive diagnoses for many severe psychiatric conditions, especially SSPD, typically necessitate prolonged periods of behavioral observation. One possible reason might be that many individuals went through an early phase of unnoticed or undeclared symptoms before their clinical visit for COVID-19. When diagnosing SSPD, doctors likely factored in this extended course of symptoms, likely triggered by the brain inflammation caused by the coronavirus infection. There also exists a possibility that some individuals could have experienced acute and transient psychotic disorders (ATPD), which were erroneously identified as SSPD. Delirium, a very common condition in critical illness, could have conceivably been mis-labelled as SSPD, although the characteristics and time course of delirium and SSPD differ significantly making misdiagnosis seemingly less likely. Conversely, patients manifesting early signs hinting at SSPD may have exhibited more distinct symptoms post-COVID-19 infection, subsequently leading to accurate diagnostic coding. Absent direct clinical assessments, pinpointing the primary factor influencing diagnosis remains elusive. Nonetheless, the sustained elevated hazard ratios even beyond the initial 90 days post-infection underscore the continued influence of COVID-19 on the emergence of SSPD.

## Conclusion

In this study, we have found a substantial increase in the likelihood of being diagnosed with a schizophrenia spectrum and psychotic disorder (SSPD) after experiencing moderate to severe illness due to SARS-CoV-2 infection, in comparison to a group of individuals who had non-COVID-19 Acute Respiratory Distress Syndrome (ARDS). Our work is consistent with the known neurotropism of the SARS-CoV-2 virus [50, 51] and other reports of increased risk of major psychiatric disorders following COVID-19 infection [33, 53–55]. Further research is required to identify specific characteristics of populations and individuals who may be at a particularly high risk of developing SSPD and potentially other significant psychiatric conditions following COVID-19 infection. Understanding these psychiatric risks associated with COVID-19 is an essential component of our strategy to address the evolving landscape of Long-COVID.

## Supporting information

S1 Appendix (ZIP)
