# Supplementary material for: SARS-CoV-2 infection is associated with an increase in new diagnoses of schizophrenia spectrum and psychotic disorder: A study using the US national COVID cohort collaborative (N3C)
Source: PLoS One. 2024 May 30;19(5):e0295891. doi: 10.1371/journal.pone.0295891 (PMC11139284; doi:10.1371/journal.pone.0295891)
Supplement: S1 Appendix — (ZIP) [file pone.0295891.s001.zip › S1_Appendix.pdf]

A Appendix A: Tables in Appendix

Table 4. SSPD Patient Comparison Among ARDS, COVID Positive and COVID Negative Group.

	**ARDS Patient N = 637**		**COVID Positive Patient N =1229**		**COVID Negative Patient = 707**		**P Value**
	**Count**	**Percentage**	**Count**	**Percentage**	**Count**	**Percentage**	
**Gender:**							
Female	222	35%	518	42%	244	35%	<2.2e-16
Male	415	65%	711	58%	463	65%	<2.2e-16
**Age Groups:**	637	Total	1229	Total	707	Total	
21 and Younger	#	#	62	5.04%	28	3.96%	4.91E-8
22 to 29	<65	<10.20%	112	9.11%	73	10.33%	0.0001715
30 to 39	94	14.76%	181	14.73%	116	16.41%	1.52E-07
40 to 49	113	17.74%	170	13.83%	112	15.84%	0.0002312
50 to 59	164	25.75%	263	21.40%	156	22.07%	1.15E-08
60 and Older	190	29.83%	441	35.88%	222	31.40%	<2.2e-16
**Race:**	637	Total	1229	Total	707	Total	
Black/African American	246	38.62%	361	29.37%	279	39.46%	6.98E-06
Asian and White/Caucasian	297	46.62%	711	57.85%	319	45.12%	<2.2e-16
**Ethnicity:**							
Hispanic or Latino	73	11.46%	118	9.60%	88	12.45%	0.003535
Not Hispanic or Latino	507	79.59%	1015	82.59%	571	80.76%	<2.2e-16

The (#) marked group has a count or proportion that is too small to quantitatively report

Table 5. SSPD Patient Comparison Within COVID Positive Group.

**Characteristics**	**COVID Positive**					
	**All N = 219264**		**Only SSPD Patient = 1229**		**Adjusted Value**	
	**Count**	**Percentage**	**Count**	**Percentage**	**Adjusted Count**	**Adjusted Percentage**
**Gender:**						
Female	103528	47.20%	518	42%	1097.457627	42%
Male*	115736	52.80%	711	58%	1346.590909	58%
**Age Groups:**					2444.048536	**Total**
21 and Younger*	7449	3.40%	62	5.04%	1823.529412	23.15%
22 to 29	19160	8.74%	112	9.11%	1281.464531	16.27%
30 to 39	30298	13.80%	181	14.73%	1311.594203	16.65%
40 to 49	34407	15.70%	170	13.83%	1082.802548	13.75%
50 to 59	53059	24.20%	263	21.40%	1086.77686	13.80%
60 and Older	74891	34.20%	441	35.88%	1289.473684	16.37%
**Race:**					7875.641237	**Total**
Black/African American*	47047	21.46%	361	29.37%	1682.451676	58.69%
Asian and White/Caucasian	131641	60.04%	711	57.85%	1184.256455	41.31%
**Ethnicity:**					2866.708131	**Total**
Hispanic or Latino	38196	17.40%	118	9.60%	678.1609195	33.47%
Not Hispanic or Latino*	165071	75.30%	1015	82.59%	1347.941567	66.53%
					2026.102487	**Total**

The (*) marked group has a significantly higher likelihood of having SSPD.

Table 6. SSPD Patient Comparison Within COVID Negative Group.

**Characteristics**	**COVID Negative**					
	**All N = 213183**		**Only SSPD Patient = 707**		**Adjusted Value**	
	**Count**	**Percentage**	**Count**	**Percentage**	**Adjusted Count**	**Adjusted Percentage**
**Gender:**						
Female	105959	49.70%	244	35%	490.945674	35%
Male*	107196	50.30%	463	65%	920.4771372	65%
**Age Groups:**			707	**Total**	1411.422811	**Total**
21 and Younger	7840	3.68%	28	3.96%	760.8695652	17.12%
22 to 29	19644	9.21%	73	10.33%	792.616721	17.83%
30 to 39	29400	13.80%	116	16.41%	840.5797101	18.91%
40 to 49	31594	14.80%	112	15.84%	756.7567568	17.03%
50 to 59	51115	24.00%	156	22.07%	650	14.63%
60 and Older	73590	34.50%	222	31.40%	643.4782609	14.48%
**Race:**			707	**Total**	4444.301014	**Total**
Black/African American*	45269	21.20%	279	39.46%	1316.037736	72.07%
Asian and White/Caucasian	133309	62.53%	319	45.12%	510.1334269	27.93%
**Ethnicity:**					1826.171163	**Total**
Hispanic or Latino	31002	14.50%	88	12.45%	606.8965517	45.29%
Not Hispanic or Latino*	166149	77.90%	571	80.76%	732.9910141	54.71%
					1339.887566	**Total**

The (*) marked group has a significantly higher likelihood of having SSPD.

Table 7. SSPD Patient Comparison Within ARDS Group.

**Characteristics**	**ARDS**					
	**All N = 222337**		**Only SSPD Patient = 637**		**Adjusted Value**	
	**Count**	**Percentage**	**Count**	**Percentage**	**Adjusted Count**	**Adjusted Percentage**
**Gender:**						
Female	105175	47.30%	222	35%	469.3446089	37%
Male*	117162	52.70%	415	65%	787.4762808	63%
**Age Groups:**			637	**Total**	1256.82089	**Total**
21 and Younger	7896	3.55%	#	#	422.5352113	11.31%
22 to 29	19885	8.94%	<65	< 10.20%	682.3266219	18.27%
30 to 39	31070	14.00%	94	14.76%	671.4285714	17.98%
40 to 49	35871	16.10%	113	17.74%	701.863354	18.79%
50 to 59	52529	23.60%	164	25.75%	694.9152542	18.60%
60 and Older	75086	33.80%	190	29.83%	562.1301775	15.05%
**Race:**			637	**Total**	3735.19919	**Total**
Black/African American*	48251	21.70%	246	38.62%	1133.640553	70.38%
Asian and White/Caucasian	138433	62.26%	297	46.62%	477.0111823	29.62%
**Ethnicity:**					1610.651735	**Total**
Hispanic or Latino	34320	15.40%	73	11.46%	474.025974	41.92%
Not Hispanic or Latino*	171609	77.20%	507	79.59%	656.7357513	58.08%
					1130.761725	**Total**

The (*) marked group has a significantly higher likelihood of having SSPD.
The (#) marked group has a count or proportion that is too small to quantitatively report.

Table 8. Comparison of SSPD Hazard Ratios Across Various Time Stages of COVID-19 Infection.

Time Frame (Semi-Annual)	Hazard Ratio	95 % Confidence Interval	
		Lower 95% CI	Upper 95% CI
January 2020 to June 2020	*	*	*
July 2020 to December 2020	0.93	0.8	1.1
January 2021 to June 2021	1.07	0.89	1.2
July 2021 to December 2021	0.88	0.727	1.082
January 2022 to June 2022	1.6053	1.2918	1.995
July 2022 to December 2022	1.5797	0.9026	2.765

*January 2020 to June 2020 time frame is considered as references.

B Appendix B: Supporting Information

S1 Table Schizophrenia Spectrum and Psychotic Disorders Code Set: list of diagnosis codes used to evaluate outcomes

S2 Table Exclusion Code Set: list of diagnosis codes used to exclude patients if one or more codes were included in the patient record prior to the index date.

S3 Table Matching Criteria Code Set: diagnosis and drug exposure codes used in the R MatchIt package to build case and control cohorts.
